# Non-Invasive *in Vivo* Quantification of Directional Dependent Variation in Mechanical Properties for Human Skin

**DOI:** 10.3389/fbioe.2021.749492

**Published:** 2021-10-22

**Authors:** Piyush Lakhani, Krashn K. Dwivedi, Atul Parashar, Navin Kumar

**Affiliations:** ^1^ Department of Mechanical Engineering, Indian Institute of Technology Ropar, Rupnagar, India; ^2^ Department of Biomedical Engineering, Indian Institute of Technology Ropar, Rupnagar, India; ^3^ Department of Plastic Surgery, Post Graduate Institute of Medical Education and Research, Chandigarh, India

**Keywords:** human skin (*in vivo*), natural skin tension, pressure, elasticity, mechanical properties

## Abstract

Skin is the body’s largest organ, and it shows non-linear and anisotropic behavior under the deformation. This behavior of the skin is due to the waviness and preferred orientation (in a particular direction) of collagen fibers. This preferred orientation of collagen fibers results in natural pre-tension and anisotropy of the skin. The knowledge of natural skin pre-tension and anisotropy is essential during incisions and surgery. The available suction-based devices quantify the anisotropy through the displacement field and cannot measure the stress-strain relation in particular directions. Therefore, in the current study, an *in vivo* full-field measurement suction apparatus was developed to measure the stress and strain of skin in all planar directions through a single experiment. First, this apparatus was tested on silicone substrates of known properties, and then it was used to test the skin of 12 human forearms. Further, to check the effect of hand stability on the measurements, the obtained results of the skin were compared with the results of a standard test performed in the same skin using a steady setup. The consistency between these two results confirms that the stability of the hand does not influence the measurements of skin properties. Furthermore, using the developed apparatus, the skin’s anisotropy and its relation with the Kraissl’s lines orientation was quantified by measuring the toe and linear moduli at an interval of one degree. The minimum and maximum values of the toe and linear moduli were 0.52 ± 0.09 and 0.59 ± 0.11 MPa, and 3.09 ± 0.47 and 5.52 ± 1.13 MPa, respectively. Also, the direction of maximum moduli was found almost similar to Kraissl’s lines’ orientation. These results confirm the contribution of skin pre-tension on the anisotropy of the skin. The present apparatus mimics the tissue expansion procedure, where observation of the test may be helpful in the selection of size and shape of the expander.

## Introduction

Understanding the anisotropic and non-linear mechanical behavior of skin is crucial for improving skin treatments such as cosmetic, reconstructive surgery, skin grafting, healing, and tissue expansions. The skin is one of the largest organs of the body, which provides protection against biological assailants and external chemicals and prevents excessive water loss. The three-layered skin structure comprises the epidermis, dermis, and subcutaneous tissue. Among these layers, the dermis is the main contributor to mechanical strength for the skin. The main constituents of the dermis are elastin fibers, collagen fibers, and ground substances [e.g., water contents and proteoglycans (PGs)]. The elastin fibers are the primary source of resistance against the deformation at small strain levels and provide resilience to the skin. On the other hand, collagen fibers are the main to provide mechanical strength of the skin at moderate and large strain levels. Also, collagen fibers are the main contributors to the skin’s anisotropic behavior.

The lack of knowledge about the capability of *in vivo* stretching, laxity, and anisotropic behavior of human skin leads to complications such as implant extrusion, necrosis, flap failure, and suture failure in tissue expansion surgery (Hodges, 1993) due to extensive stretching of the skin above the sustainable capacity of the pressure. Tissue expansion is a widely used technique for hair transplant, traumatic defect repair, burnt skin replacement, and removal of pigmented strains. Therefore, *in vivo* qualitative and quantitative knowledge of the non-linear and anisotropic behavior of the skin is helpful for many medical applications (Pamplona et al., 2014a).

Dupuytren (1836) observed noncircular wound formation upon puncturing with a round tool indicating the anisotropic nature of the skin. In 1861, Langer (1861) discovered skin tension lines on the cadaveric skin; these lines are known as Langer’s lines. These lines show subject-specific variability at different body sites. Subsequently, several theories have since been developed on the cause of skin tension lines (Wilhelmi et al., 1999). Relaxed skin tension lines (Borges, 1989) (RSTLs) and Kraissl’s lines (Kraissl, 1946) are universally accepted guidelines to define the direction of skin tension lines (STLs) on living subjects (Laiacona et al., 2019). The RSTLs measurement has limitations of angular resolution because it is measured by manual pinching on the skin to observe the furrows. On the other hand, Kraissl’s lines measurement has better accuracy, as these lines are determined based on the formation of wrinkles. Excluding the face, RSTLs and Kraissl’s lines are consistent with each other for the remaining body parts (Piérard and Lapière, 1987; Wilhelmi et al., 1999). However, Langer lines are not consistent with RSTLs and Kraissl’s lines; as, in the ventral forearm, the direction of Langer lines deviates about 40–60° from the direction of RSTLs and Kraissl’s lines (Wilhelmi et al., 1999). Currently, RSTLs (for the face) and Kraissl’s lines (for the rest of the body) are generally followed by surgeons for incision planning (Son and Harijan, 2014). These skin tension lines (STLs) are due to the arrangement of the collagen and elastin fibers in the dermis (Piérard and Lapière, 1987).

The collagen fibers in the STLs direction are taut and take an early load compared to other directions, which results in the anisotropic behavior of the skin (Pissarenko et al., 2019). Experimental methods such as uniaxial (Ní Annaidh et al., 2012) and biaxial (Aldieri et al., 2018) tensile tests are widely reported for the *in vivo* (Coutts et al., 2013; Jacquet et al., 2017; Khatyr et al., 2006) and *ex vivo* (Dwivedi et al., 2020b, 2020a; Meador et al., 2020; Ottenio et al., 2015) studies to quantify the mechanical anisotropy of the skin. However, in these methods, the directions of applied load are limited, which fails to explain the complete anisotropy of tissue. Therefore, Kvistedal and Nielsen, 2009 developed a multiaxial testing rig to apply simultaneous loads in multiple directions for *in vivo* testing of the skin. However, due to the bulky size of the setup, it was not easy to implement on all the body sites for *in vivo* studies. A review article published by Pissarenko and Meyers (2020) concluded that the uniaxial tensile loading provides limited information for the anisotropic characterization of the skin. Therefore, the data from these experiments do not accurately reflect the anisotropic behavior of skin in numerical modeling while considering collagen fibers dispersion. Hence, attention needs to be paid to the *in vivo* experimental technique with multi-directional loading so that the study’s observation may be directly used in the clinical setup.

The shear wave or surface wave technique (Deroy et al., 2016; Gahagnon et al., 2012; Zhang et al., 2018) has been previously used to measure elasticity and anisotropy in an *in vivo* setup. However, this technique applies a small deformation and requires multiple tests to measure the anisotropy. In contrast, surgical procedures such as skin grafting and tissue expansion involve large deformations and higher loadings. Therefore, understanding the mechanical behavior of skin at extensive stretching is essential. The bulge test method (Diab et al., 2020; Lakhani et al., 2020; Tonge et al., 2013) can apply load in all directions and overcome the limitation of small deformation but can only be used in *ex vivo* tests.

The suction method (a technique similar to the bulge test) is a widely used *in vivo* technique to measure the mechanical properties of human skin (Escoffier et al., 1989; Regoire et al., 1998). Previous studies have used this method to quantify the effect of age and disease on the mechanical properties of the skin (Diridollou et al., 1998; Grahame and Holt, 1969). Further, several studies have confirmed the clinical applicability of the suction method. (Elrod et al., 2018; Khatyr et al., 2006; Laiacona et al., 2019; Neto et al., 2013; Pedersen et al., 2003). However, the commercially developed [Cutometer^®^ (Barbarino et al., 2011; Dobrev, 2007; Elrod et al., 2018; Weickenmeier et al., 2015)**,** Cutiscan^®^ (Rosado et al., 2016)] and the conventional suction test methods are not capable of measuring the anisotropic stress-strain relations. Moreover, the straining system developed by Laiacona et al., (2019) measures the anisotropy of skin in terms of the strain field and extends the capability of the suction method to determine the STLs direction. However, due to the use of a single camera in the straining device, it is not capable of measuring the stress-strain response for the skin. In conclusion, to date, no system is available that can measure the stress-strain relation for *in vivo* skin in all the direction through a single test. Therefore, in the current article, a novel apparatus was developed to measure the mechanical properties in all the planar directions through a single test.

The conventional method for the mechanical anisotropic properties such as extensometry, shear or surface wave, and bulge test has been used to investigate the variation of the in-plane mechanical properties at an interval of 3° (Ruvolo et al., 2007), 10° (Deroy et al., 2016; Lakhani et al., 2020), 22.5° (Kvistedal and Nielsen, 2009), 30° (Flynn et al., 2011, 2013), 45° (Jacquet et al., 2017; Khatyr et al., 2006; Ní Annaidh et al., 2012; Ottenio et al., 2015; Vexler et al., 1999) and 90° (Meijer et al., 1999; Gahagnon et al., 2012; Boyer et al., 2013; Tonge et al., 2013). Therefore, to increase the angular resolution of calculated properties at the one-degree interval and accurate measurement of skin pre-tension direction, a full-field measurement suction apparatus was developed in the current study, where the suction test method was coupled with the Digital Image Correlation (DIC). The developed apparatus overcomes the limitation of conventional methods and measures the mechanical properties in 360° angular directions in a single test. The developed apparatus was validated by measuring the properties of specimens with known properties. Moreover, the applicability of the apparatus was confirmed by performing *in vivo* test on human skin. This apparatus requires minimum preparation, and its compactness allows the testing of individual subjects at different body sites.

The objectives of the current study are; to

• validate the developed apparatus and test the capabilities of the full-field measurement suction apparatus for non-invasive *in vivo* testing on human skin.
• measure the consistency between the maximum linear modulus orientation and the direction of Kraissl’s line.
• study the directional dependent variations in the toe modulus and linear modulus at an interval of one degree to quantify the *in vivo* mechanical anisotropy of the skin.

## Materials and Methods

### Full-Field Measurement Suction Apparatus

**FIGURE 1:**
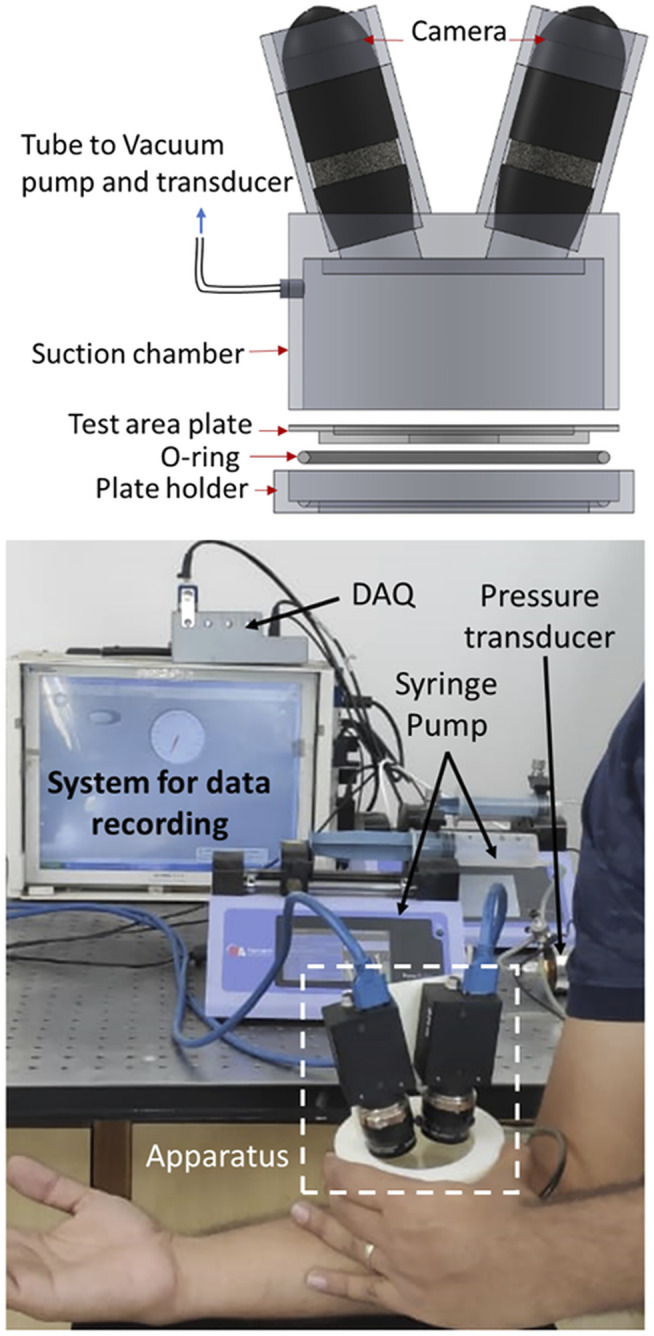
The schematic diagram and photograph of full-field measurement suction apparatus. The apparatus consists of a suction chamber, two cameras, a vacuum pump, pressure transducer, test area plate, O-ring, and plate holder.

The apparatus consisted of two cameras, a test area plate, O-ring, a plate holder, and a suction chamber (Figure 1). The suction pump and pressure transducer were connected with the suction chamber through a flexible tube. The suction chamber had dimensions of 100 mm outer diameter, 84 mm inner diameter, and 30 mm depth. The cameras and suction chamber were isolated using an acrylic plate. A circular plate with a 30 mm diameter hole was chosen for the suction test based on the available flat surface area on the forearm skin. Suction was applied using two syringe pumps, where one of the pumps (Harvard apparatus, pump 11 elite) was programmed to create the vacuum, and the second pump was attached for safety (to release the vacuum in case of failure in the first syringe pump). The pressure transducer (Range: 0–2 bars absolute pressure, accuracy: ± 0.1%) recorded the vacuum through a data acquisition system (NI cDAQ 9174, module: NI9203). Images were captured using two stereoscopic digital cameras (5 MP resolution, Flir Systems Inc., Canada). The camera angle was set to maximum (nearly 28° angle) to capture the deformed skin’s curvature by keeping the compactness of the setup. Further, to accommodate the out-of-plane displacement during the test, the depth of field was set to maximum by minimizing the lens aperture. The images were captured using Vic-snap 8 (Correlated Solutions, United States).

The institute’s ethical norms were followed for non-invasive testing on human skin. 12 male volunteers from Indo-Aryan ethnicities of the age range from 26 to 31 years had participated in the experiment with signed informed consents. The tests were performed on the skin of the upper volar forearm of the right hand. The skin was washed using the liquid body wash approximately 1 h before the actual test. Then, all the subjects were instructed to stay in the environment temperature 26–28°C and Relative Humidity of 40–50% until the test. The liquid body wash and uniform environmental conditions for all the subjects were essential to maintain uniform hydration of the skin as an alteration in hydration level may affect the mechanical properties of the stratum corneum. However, it is well reported that the stratum corneum is an ultrathin layer of skin and does not contribute significantly to the skin’s mechanical properties (Wu et al., 2006). Further, based on the skin color, white or black nonuniform speckles were generated (using the airbrush with 0.25 mm nozzle diameter) to achieve high contrast for DIC.

During the experiments, the forearm was kept relaxed and placed on a flat table such that the elbow remained bent nearly 90° (as shown in Figure 1). To keep the consistency among the measurements, X-axis was taken along the long axis of the arm, which was directed toward the elbow. The syringe pump was set to withdraw 40 ml air at a 40 ml/min flow rate and inject it back at the same flow rate to release the vacuum. The experiment consisted of single loading and unloading cycle. The experiments were performed without preconditioning, as it induces the inelastic deformation on the specimens (Dwivedi et al., 2020a), which alters the mechanical response of the skin. In contrast, the objective of the test was to measure the mechanical response of the skin under its natural state. During the test, the specimen (*in vivo* skin) got attached to the boundary of the test area due to the vacuum inside the chamber, where maximum pressure was reached in a range of 17–26 kPa for different subjects. After unloading, the specimen returned to its initial condition (nearly zero strain). The test was considered to be a failure if the sudden change (rise/fall) in the pressure was observed during loading or if any visible slip of skin occurred from the boundary. The recorded videos were watched carefully to find out the slipping. The pressure value was recorded with a sampling rate of 100 data points per second, whereas the images were captured with a frame rate of 4 images per second. Three trials were performed on the same subject at the same location to confirm the repeatability of the observations. Although no study reported the complete restoration of skin after stretching, we had kept a gap of at least 15 days between the tests to avoid the unrecovered effect of the previous test. Similarly, for the failed tests, the tests were repeated after 15 days following the same test protocols. Participants did not feel any discomfort during or after the test, and no visible dent appeared on the test area after the test.

### Full-Field Stress-Strain Calculations

**FIGURE 2:**
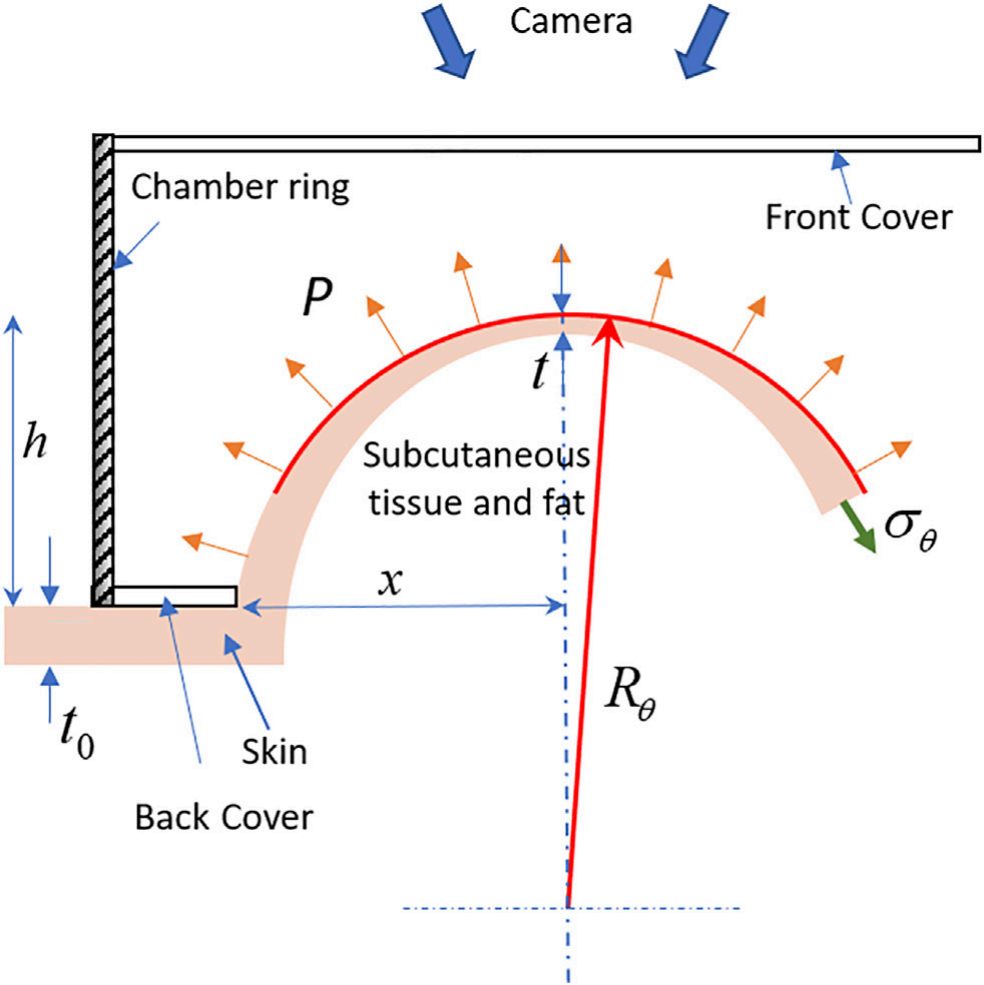
A schematic diagram of the front cross-sectional view of the specimen deformation illustrates the thickness variation near the apex and deformed geometry of the skin.

The in-plane stresses were calculated using the membrane theory (Eq. 1) with the assumption of uniform stresses along the thickness direction. Moreover, the out of plane stresses was considered negligible because of the smaller stress value than in-plane stresses for thin membranes (Tonge et al., 2013). Also, the bending stresses were considered negligible due to the low rigidity of the skin in the bending. The apparatus was placed on the arm vertically, and the skin took a hemispherical shape within the test region due to the weight of the apparatus. The radius of curvature was calculated using the coordinate points on the surface of the specimen, and the initial value of strains was considered to be zero. Further, the center of the test area was taken as the origin of the materials coordinate systems. The anisotropic properties of the skin led to a nonuniform deformation of the skin, which resulted in a varying radius along the different directions. Therefore, the Cauchy stresses ( $\sigma_{\theta }$ , in kPa) in the localized directions at an interval of 1° were calculated using Eq. 1 as a function of pressure (*P*, in kPa), radius [ $R_{\left(\theta +90\right)}$ , in mm], and thickness (*t,* in mm) (Figure 2).

$$\sigma_{\theta }=\frac{P\cdot R_{\left(\theta +90\right)}}{2\cdot t}$$ (1)

**FIGURE 3:**
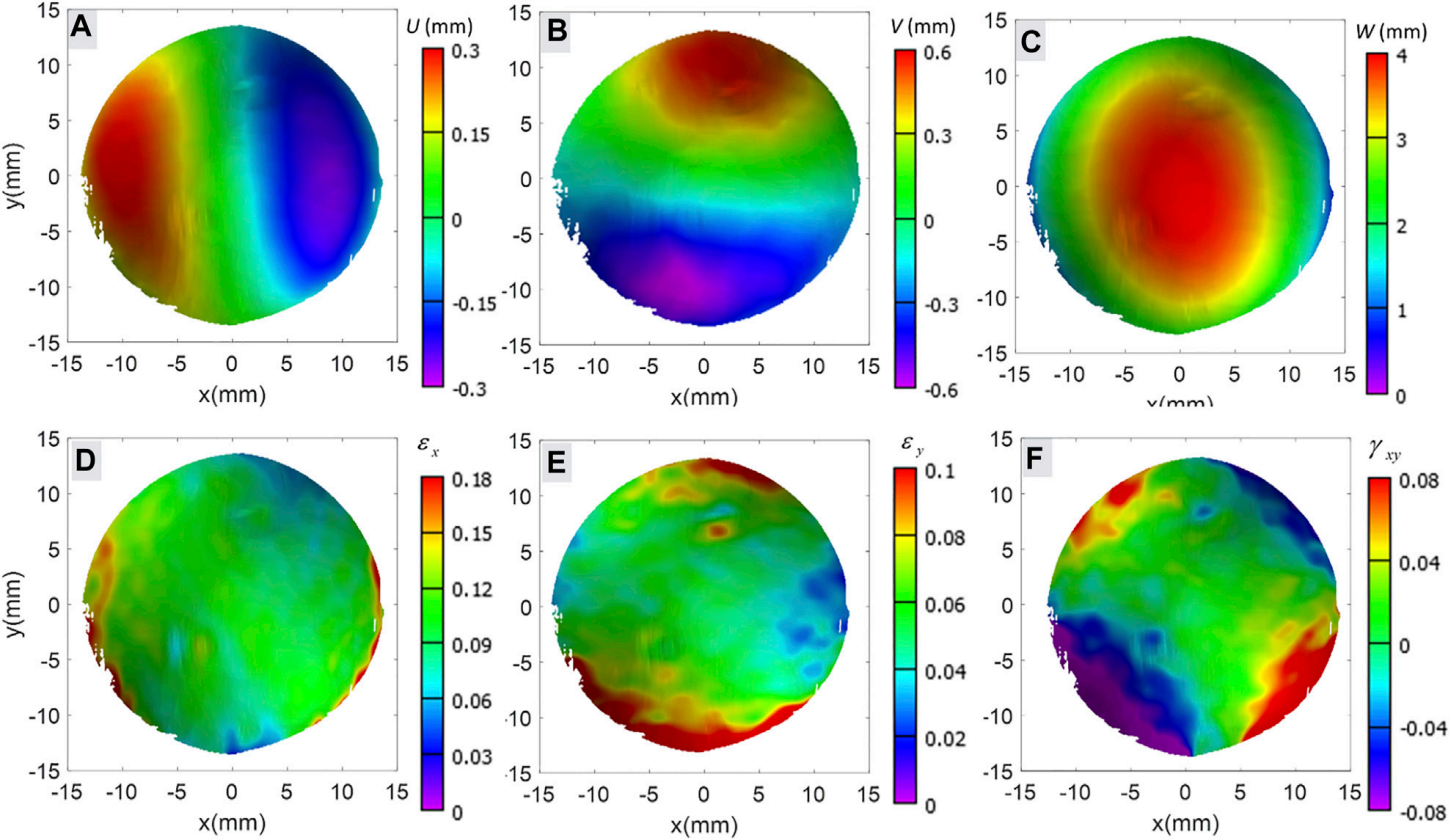
Shows the representative full-field displacement and calculated Green-Lagrangian strains obtained through DIC in **(A)** displacement in *X*-axis direction, **(B)** displacement in *Y*-axis direction, **(C)** out of plane displacement, **(D)** normal strain in *X*-axis direction, **(E)** normal strain in *Y*-axis direction, and **(F)** shear strain on *XY*-plane.

The radius of the curvature was calculated using the full-field displacement obtained from the DIC (analyzed using the Vic-3D software). The representative full-field deformed coordinates *U*, *V*, and *W* corresponding to the *X*, *Y*, and *Z*-axis are shown in Figures 3A–C. The refractive index of the acrylic plate attached to the vacuum chamber may affect the obtained displacement field. Therefore, preliminary tests were performed on a rigid plastic ball of nearly 40 mm diameter to quantify the shift, which reported 0.23 mm lateral and 0.06 mm normal shift without any deformation. However, the radius of curvature and strain did not alter due to consistent shift. The radius of curvature with and without acrylic plate was found 39.84 and 39.91 mm, respectively. However, almost zero strain ( $3\times 10^{-10}$ ) appeared due to the acrylic plate. The error in the displacement and strain was calculated by simulating the rigid body motion, where the same image is translated by 2, 5, and 10 pixels in a single direction (Lakhani et al., 2020). The mean error in displacement and strain was found ± 3.074 µm and $\pm 0.941\times 10^{-6}$ , respectively.

The primary calculation showed that the 20 mm diameter area from the center of the material coordinate system showed a good circular fit for the curvature measurement. Therefore, it was defined as the region of interest for further analysis. For each direction at an interval of 1°, one hundred data points at a 0.2 mm distance were extracted within the plane. The coordinate points (*X*, *Y*, and Z) were used for the calculation of the radius of curvature using the Gaussian elimination algorithm in MATLAB (MathWorks inc., United States) as described by Lakhani et al., (2020) for the bulge test.

The thickness of the skin was estimated based on the observation of Van Mulder et al., (2017). This study reported the effect of BMI on the skin thickness of the ventral forearm, dorsal forearm, and deltoid. Further, they derived the mathematical model for the skin thickness variation as a function of gender and BMI. This model was used to estimate the skin thickness (*t*_0_, in mm) for the forearm skin (Table 1). Moreover, in the absence of a study that has evidence about the significant variation in the skin thickness within the small planar area, we have assumed uniform thickness for the skin on the forearm. Further, the thickness of the skin cannot be considered constant for the large finite deformation as described schematically in Figure 2. Therefore, the relation derived by Slota (Slota and Spisak, 2005), which measures the thickness (Eq. 2) in terms of the test area (*x*, in mm) and inflation height (*h*, in mm), was used to calculate the thickness of the specimen during deformation.

$$t=t_{0}\left[\frac{x^{2}}{x^{2}+h^{2}}\right]$$ (2)

**TABLE 1:** The calculated thickness for individual subjects.

Subject	S1	S2	S3	S4	S5	S6	S7	S8	S9	S10	S11	S12
BMI	27	22	25	20	22	24	21	27	26	22	23	21
Thickness (mm)	1.25	1.15	1.20	1.10	1.15	1.20	1.10	1.25	1.25	1.15	1.20	1.10

Full-field Green-Lagrangian strains corresponding to the *X*, *Y* directions ( $\varepsilon_{x}$ , $\;\varepsilon_{y}$ ) and shear strain on *XY* planes ( $\gamma_{xy}$ ) (Figures 3D–F) were calculated using full-field displacement obtained from the DIC using the Vic-3D software. A mean of 100 data points along the angle *θ* within the ROI was used to calculate $\varepsilon_{x\theta }$ , $\varepsilon_{y\theta }$ , and $\gamma_{xy\theta }$ . Further, the normal strain ( $\varepsilon_{\theta }$ ) in the particular direction of angle *θ* was calculated using Eq. 3 (Hibbeler, 2014).

$$\varepsilon_{\theta }=\frac{\varepsilon_{x\theta }+\varepsilon_{y\theta }}{2}+\frac{\varepsilon_{x\theta }-\varepsilon_{y\theta }}{2}\cos\left(2\theta \right)+\frac{\gamma_{xy\theta }}{2}\sin\left(2\theta \right)$$ (3)

### Apparent Modulus for Toe and Linear Regions

The typical stress strain curve of the skin shows two distinct linear regions. The slope of the initial linear region is known as the toe modulus, and the slope of the second linear region is known as the linear modulus. Further, to measure the variation of toe modulus and linear modulus with the directions, the mean value of stresses and strains in the particular directions were obtained. In the moduli measurements, the role of subcutaneous tissue was not considered; therefore, the moduli were redefined as apparent moduli throughout the article. The apparent moduli were calculated using the Generalized Hooke’s law relation. Further, during the experiment, the induced strain on the skin was large (∼10%); therefore, considering the finite strain theory, the Green-Lagrangian strain and second Piola-Kirchhoff stress were used in Generalized Hooke’s law equation. The Cauchy stress ( $\sigma_{\theta }$ ) was transformed into the second Piola-Kirchhoff stress ( $S_{\theta }$ ) using relation $S_{\theta }=J\;\lambda_{\theta }^{-2}\;\sigma_{\theta }$ (Holzapfel, 2000). Here, the skin was considered an incompressible material, and the value of the determinant of deformation gradient tensor (*J*) was taken equal to one. The value of the stretch ratio $\left(\lambda_{\theta }\right)$ was calculated using relation $\lambda_{\theta }=\sqrt{2\;\varepsilon_{\theta }+1}$ , where $\varepsilon_{\theta }$ denoted Green-Lagrangian strain.

The orthogonal coordinates with one principal axis in the direction of *θ* and another at *θ +* 90° on the plane parallel to the epidermis were taken in stress and strain calculation calculations. In order to quantify the toe and linear moduli in all the directions, the stress-strain relations calculated at an interval of 1° on the plane of the skin surface were used in Eqs 4, 5. The toe region was considered up to 5.0 ± 0.3 kPa, and the linear region was taken based on the data points corresponding to 20 ± 1% of the maximum strain value in the direction of maximum strain. Here, the toe moduli and linear moduli in the direction of angle *θ* were defined as $E_{\theta }^{T}$ and $E_{\theta }^{L}$ , respectively. Similarly, the moduli in the direction of angle *θ +* 90° were defined as $E_{\theta +90}^{T}$ and $E_{\theta +90}^{L}$ , respectively. Similarly, the moduli were calculated for all the directions at an interval of 1°. The Poisson’s ratio ( ν ) was taken as 0.5 (limit of incompressible material) independent of the direction (Laiacona et al., 2019).

$$\varepsilon_{\theta }=\frac{1}{E_{\theta }}\cdot S_{\theta }-\frac{\nu }{E_{\theta +90}}\cdot S_{\theta +90}$$ (4)

$$\varepsilon_{\theta +90}=-\frac{\nu }{E_{\theta }}\cdot S_{\theta }+\frac{1}{E_{\theta +90}}\cdot S_{\theta +90}$$ (5)

The direction of maximum linear modulus represents the mean orientation of collagen fibers, which depicts the orientation of the STLs. Further, to understand the contribution of STLs in the anisotropy of skin, the coordinate system was re-oriented such that the direction of maximum linear modulus (representing STLs) was taken as 0° for each subject. Then, the quadratic polynomial interpolation was taken to calculate the stresses corresponding to the common strain values for each curve. Further, the mean of three curves in the corresponding direction was calculated to obtain each subject’s mean stress strain response.

### Orientation of Kraissl’s Lines

**FIGURE 4:**
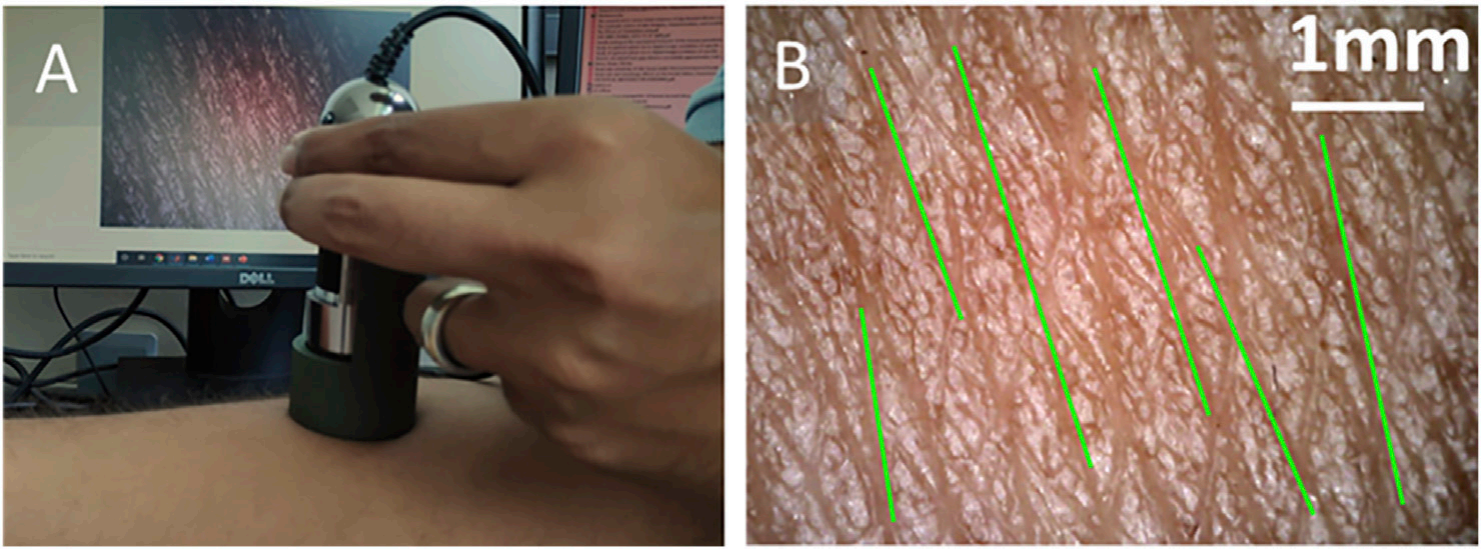
**(A)** The hand-held microscope used to capture the magnified image of the skin surface. **(B)** The captured image of the skin and identified orientation (green lines) of the wrinkle lines.

The subject-specific orientation of Kraissl’s lines was measured using the image processing technique, which was developed based on the observations of Kraissl (1946). The study reported the direction of skin tension line in the direction of wrinkle lines. Conventionally, the orientation of these lines has been measured qualitatively through eye observation; hence, this technique has a limitation of angular resolution. The current study introduces an image processing method for the microscopic image to measure the orientation of primary microrelief lines (wrinkle lines). In this method, the images were captured using the hand-held digital microscope [Dino-Lite Edge, 5 MP (2592 × 1944 pixels) resolution] at the same location where the suction test was performed, as shown in Figure 4A. The reference direction was taken similar to the suction test. The brightness, contrast, and gamma for the microscope were adjusted manually to increase the contrast between the primary relief lines and other skin areas.

The image processing algorithm was implemented in MATLAB. Initially, an empty mask was created on the image, which subsequently overwrote the identified regions of the primary relief lines. This algorithm requires manual identification of the primary relief line (wider) by selecting one point on an individual line. Further, based on the grayscale value with tolerance 0.02 using “*grayconnected*” function, one primary line has been identified. This identified region was overwritten on the empty mask. This process was repeated 4–7 times depending on the number of primary lines visible on each image. Then, the image was binarized to identify the individual primary line using the “*bwlabel*” function. Further, the “*regionprops*” function was used to fit the ellipse on identified region and to calculate the major axis length, orientation angle, and centroid. The “*bwlabel*” function may find more regions than the number of selected lines; therefore, these lines were filtered based on the length of the major axis. Then, the identified lines were drawn on the original RGB image using centroid, orientation, and length for representation, as shown in Figure 4B. A mean value of orientation was taken as the orientation of the microrelief line for a particular image. Further, the procedure was repeated for three different locations within the region of the suction test. This approach cannot detect all the visible primary lines due to lower contrast. However, the overall calculated results do not significantly vary from the calculated orientation.

### Isotropic Silicone Substrates

Isotropic silicone substrates of different stiffness were fabricated to validate the accuracy and repeatability of the developed apparatus. The silicone substrates of 2.5 mm thickness and 50 mm diameter were fabricated by mixing the base with a curing agent in a weight ratio of 5:1 and 18:1 (sylgard 184). These ratios have been chosen intentionally to make substrates with higher modulus differences. Five specimens of each weight ratio were used to validate the apparatus, and 15 specimens of 18:1 ratio were used to check the repeatability of the results. Suction tests were performed on each specimen following the same protocol described for the *in vivo* human skin. Similarly, the rectangular specimen of 75 × 75 mm was fabricated to cut five dumbbell shape specimens of the dimension described by Dwivedi et al. (2020a, 2020b). Then, a uniaxial tensile test with a displacement rate of 0.05 mm/s was performed on the specimens of both ratios, where the strain was measured using the 2D digital image correlation. Then, the modulus was calculated by measuring the slope of stress-strain response for each substrate.

### Quantifying the Effect of Hand Steadiness

The steadiness of the operator’s hand may affect the results obtained from the developed hand-held device. Therefore, to study the possible error that may occur due to the stability of the hand, a supplementary study was performed with the steady experimental setup as shown in Supplementary Figure S1. Cameras and a suction chamber were mounted on the test bench to make the system stable in this setup. The system used for the steady setup had similar specifications as described for the apparatus. To keep the consistency between the hand-held apparatus and steady setup, the protocol described in *Full-Field Measurement Suction Apparatus* was used for the experiments, and three repetitive tests were performed on the total five subjects (from S1 to S5).

### Statistical Analysis

The non-parametric Kruskal-Wallis test was performed to test the statistical difference in the parameters among the subjects, where the three repetitive tests performed on an individual subject were taken as a group. This method was used to compare the modulus, the maximum linear modulus orientation, and Kraissl’s lines among the groups (subjects). Also, the same statistical method was used for the analysis of the effect of hand steadiness on the measured properties. Further, paired *t*-test was performed for the measurement of statistical difference between the uniaxial and suction test method, which was used for the validation of apparatus. Also, the evidence of significant differences in the maximum linear modulus orientation and Kraissl’s lines direction was evaluated using the *t*-test. The level of significance was set to *p* = 0.05 for all statistical tests. The results were reported as mean ± standard deviation (SD).

## Results and Discussion

### Validation of Apparatus

The results of supplementary experiments performed on the silicon substrates using the developed apparatus and uniaxial tensile test (UTT) are presented in Supplementary Figure S2. The modulus calculated from the results of the UTT for the substrates of weight ratio 5:1 and 18: 1 were found 3.53 ± 0.12 and 1.15 ± 0.09 MPa, respectively. However, the modulus measured from the developed apparatus were 3.79 ± 0.21 MPa (for weight ratio of 5:1) and 1.30 ± 0.10 MPa (for weight ratio of 18:1). There is no evidence of significant difference was found between the values of moduli (*p* = 0.081, for substrates made of weight ratio 5:1, and *p* = 0.224, for 18:1 silicon substrate) calculated from UTT and developed apparatus. Moreover, the small difference between the UTT measured modulus and apparatus measured modulus could be due to the assumptions of membrane theory which was used to measure the stress in the suction test. Therefore, the consistency between the results of UTT and the developed apparatus confirms its accuracy. Furthermore, the repeated tests performed on the fifteen silicone substrates of 18:1 weight ratio showed a small variation (approximately ± 7%) in the mean value of moduli (1.28 ± 0.09 MPa), which also confirms the reproducibility of the results obtained through the developed apparatus.

### Mechanical Anisotropy

The stresses calculated for the *in vivo* suction test may be affected by the initial curvature of the skin. Therefore, in order to identify the subject-specific differences in the initial test condition, the radius of curvature at the beginning of suction was considered in statistical analysis. The initial radius of curvatures was found in the range of 32–43 mm in all the tests. However, no evidence of a statistically significant (*p =* 0.23) difference was found in the radius of curvature across the subjects. Moreover, the toe modulus and linear modulus were calculated based on the slope of the stress-strain curve. Therefore, it can be concluded that the initial curvature did not significantly affect the calculated properties.

**FIGURE 5:**
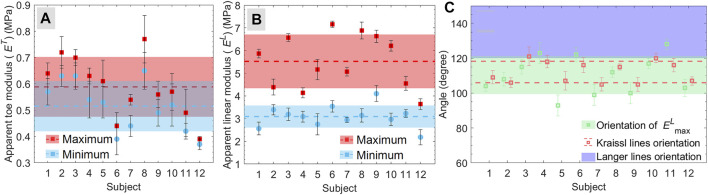
**(A,B)** maximum and minimum apparent modulus for **(A)** toe region and **(B)** linear region calculated for individual subjects. The dashed lines and shaded regions with the same color represent the mean and standard deviation, respectively, for the respective values. **(C)** the maximum linear modulus orientation and direction of Kraissl’s lines. The blue shaded region (in C) represent the estimated range of Langer lines (Langer, 1861).

The maximum and minimum apparent toe moduli were found 0.59 ± 0.11 and 0.52 ± 0.09 MPa, respectively; and the maximum and minimum apparent linear moduli were 5.52 ± 1.13 and 3.09 ± 0.47 MPa, respectively (Figures 5A,B). Also, a significant difference in the value of linear moduli (*p* = 0.00055) was observed among the subject. However, the results were found consistent with the reported moduli (0.05–80 MPa) for *in vivo* (Agache et al., 1980; Hendriks et al., 2003; Khatyr et al., 2006) and *ex vivo* studies (Ní Annaidh et al., 2012; Ottenio et al., 2015). The ratio for the maximum to minimum apparent linear modulus was found 1.85 ± 0.31, which was in good agreement with the reported value of 1.93 for the volar forearm (Liang and Boppart, 2010). These consistencies in the reported values of modulus and the current study show the appropriateness of the testing method.

The present apparatus measures the local strains and inhomogeneous stresses that overcome the limitations of global strain and stress calculation-based methods used in the conventional suction test (Elrod et al., 2018). Moreover, it can be observed from the literature that the types of loading (uniaxial, biaxial, and multiaxial) significantly affect the natural orientation of collagen (Jacquet et al., 2017; Kvistedal and Nielsen, 2009; Lakhani et al., 2020). The load applied in selected directions straightens the collagen fibers in the loading direction through sliding and rotations (Yang et al., 2015). Therefore, most of the collagen fibers participate in the mechanical response for individual direction, which may lead to the erroneous interpretation of anisotropy and collagen dispersion. Conversely, the suction test applies uniform loading in all the planar directions. Hence, the developed full-field measurement suction apparatus under radially symmetric loading can better explain the anisotropic response.

The suction test method has limitations due to a lack of control on the loading strain rate because compressible fluid air was used in the test. The suction test cannot control the strain rate accurately as compressible fluid air was used in the test. However, it can be believed that under quasi-static loading, the soft tissues show almost elastic behavior (Bose et al., 2020; Dwivedi et al., 2020b; Ní Annaidh et al., 2012; Ottenio et al., 2015; Zhou et al., 2010), and a slight change in strain rate within the quasi-static range does not affect the elastic properties of tissues. Therefore, the tests were performed under a quasi-static stretching to minimize the effect of nonuniform strain rate on the mechanical response. Further, the representation of skin as a membrane ignores the connection with the subcutaneous tissue and layer of fat. This assumption was taken in this study due to the lack of reliable calculation methods reported in the literature for the stress resistance due to these tissues (Flynn et al., 2013; Kvistedal and Nielsen, 2009). However, the softer hypodermal layer is isotopic and has a relatively small contribution [twofold smaller modulus (Guimarães et al., 2020)] on the mechanical properties. Therefore, this layer does not alter the measured properties and anisotropy significantly. The collagen fibers are the main load-carrying constituents of the skin; hence the preferred alignment of collagen in a particular direction leads to a higher stiffness (modulus) in that direction (STLs direction). Therefore, it can be hypothesized that the direction corresponding to the maximum linear modulus represents the orientation of STLs.

### Orientation of Kraissl’s Line and Maximum Linear Modulus

The mean orientation of maximum linear moduli for all the subjects was found 110.3° ± 10.4° (Figure 5C). The comparison of maximum linear modulus orientation with Langer’s lines orientation is shown in Figure 5C (Langer, 1861), where the range and standard deviation for these lines on the volar forearm were obtained from the various reproduced version of Langer’s lines (Wilhelmi et al., 1999). Moreover, the mean orientation of the Kraissl’s line on the volar forearm obtained using the image processing approach was found 112.1° ± 5.9°, as represented in Figure 5C. Statistical analysis between the maximum linear modulus orientation and Kraissl’s lines directions for each subject showed no evidence of significant difference (*p* > 0.05) was found except subjects S5 and S11 (Figure 5C). These consistencies between the orientation of maximum linear modulus and Kraissl’s lines directions show the contribution of the STLs on the mechanical anisotropy of *in vivo* human skin. Further, there is a statistically significant difference found in the orientation of the maximum linear modulus (*p* = 0.0058) across the subjects. Similarly, Kraissl’s lines’ orientation (*p* = 0.0024) across the subjects was found a statistically significant difference. These results are found in agreement with the observation of Langer (Langer, 1861), who reported that the subject-specific variation could be expected for the direction of STLs. Further, considering all the subjects, there is no evidence of a statistical difference between maximum linear modulus orientation and Kraissl’s lines were found (*p* = 0.40). In the current study, the assumption of uniform thickness has a consistent effect on the calculated stresses in each direction. Therefore, the variation in stresses along different directions cannot be affected by the thickness. Hence, the measured maximum linear modulus orientation is not affected by the assumption of the initial skin thickness.

### Anisotropy, Modulus Ratio, and Collagen Dispersion

**FIGURE 6:**
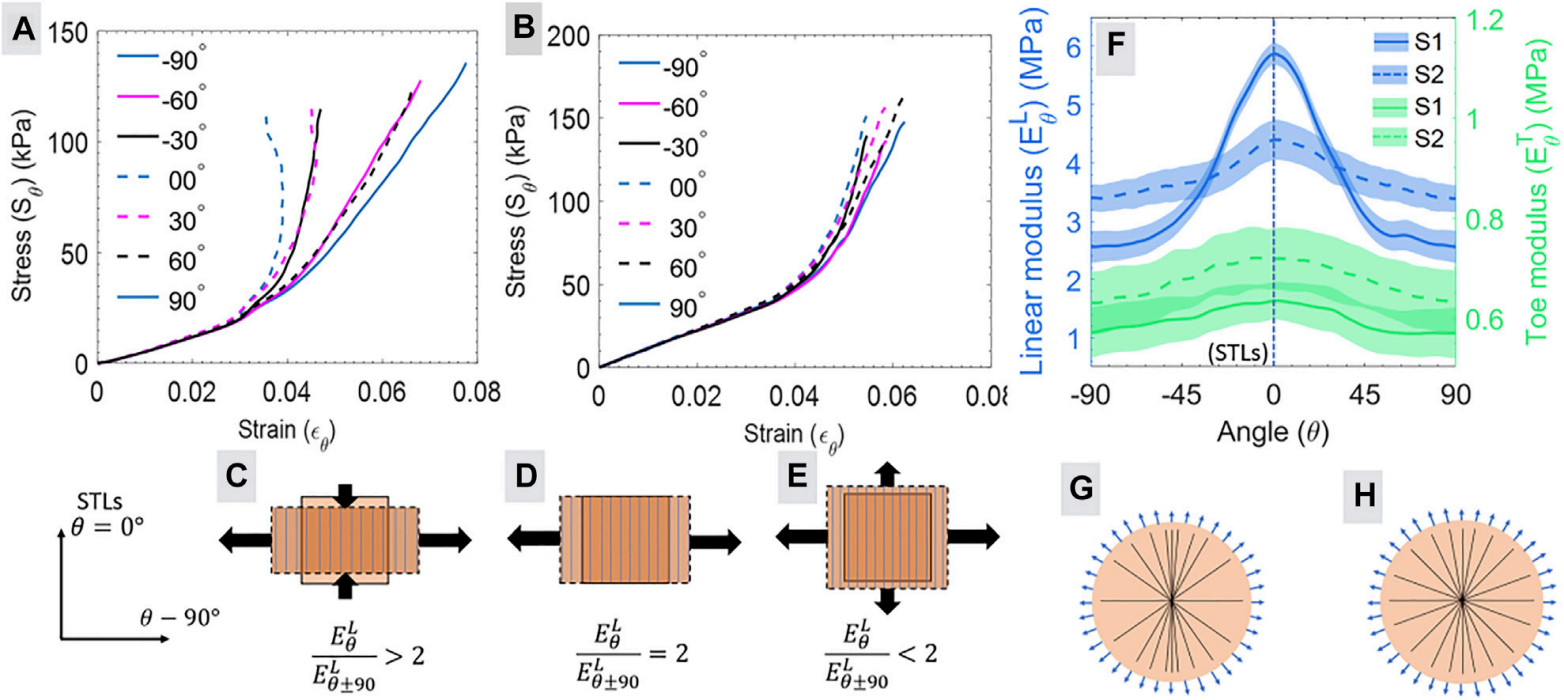
**(A, B)** illustrates the stress-strain calculated at an interval of 1° for the subjects S1 and S2, respectively, with a reduced number of angles. The wide-span width of **(A)** shows more anisotropy than the narrow span width in **(B)**. The modulus ratio calculated for the linear regions based on **(A, B)** can be characterized in three categories shown in **(C–E)**. The vertical lines represent the direction of collagen fibers, and an arrow represents the strain. (−90° and 90° represents the same direction). **(F)** shows the mean and standard deviation for the variation in the linear modulus (left *y*-axis) and toe modulus (right *y*-axis) with the angle for subjects S1 and S2. The schematic in **(G)** and **(H)** illustrates the probability of directional distribution intensity of collagen fiber (straight lines) distribution for subjects S1 and S2, respectively. The higher modulus variation (in A) for S1 shows more preferential orientations of fibers as represented in **(G)**, and the lower variation (in B) shows a more uniform distribution of fibers described in **(H)**.

The maximum and minimum apparent moduli represented in Figures 5A,B designates the anisotropic nature of the skin, where the higher linear modulus ratio compared to toe modulus ratio (Table 2) indicates the effects of the collagen fibers preferred orientation on the anisotropy of the skin. Further, to understand the correlation between the level of anisotropy and mechanical response, the subjects were divided into two groups (G1 and G2) based on the moduli ratio. The subjects (S1, S3, S6, S8, and S10) with moduli ratios greater than two were included in group G1 whereas group G2 contained subjects (S2, S4, S5, S7, S9, S11, and S12) with modulus ratios less than two. The mean stress-strain curve for subjects S1 (from G1) and S2 (from G2) are shown in Figures 6A,B, respectively, with a reduced number of lines (at the interval of 30°) for the better visualization of the variation.

**TABLE 2:** Shows the modulus ratio for toe and linear regions for each subjects.

Subject	Toe modulus ratio	Linear modulus ratio
S1	1.12	2.29
S2	1.14	1.29
S3	1.11	2.06
S4	1.17	1.34
S5	1.15	1.88
S6	1.13	2.02
S7	1.23	1.72
S8	1.18	2.19
S9	1.14	1.62
S10	1.10	2.09
S11	1.17	1.41
S12	1.05	1.67
Mean	1.15	1.85
Std. dev	0.04	0.31

The span width of stress strain curves for different directions and differences in the nonlinearity between the subjects of groups G1 and G2 (Figures 6A,B) can be explained by modeling the suction test as an equibiaxial ( $\sigma_{\theta }=\sigma_{\theta +90}$ ) loading condition as illustrated in Figures 6C–E. This simplified model helps to explain the subject-specific variation in the span width of the stress-strain relationship, modulus, modulus ratio, and anisotropy. The stress-strain conditions for the biaxial state of the stress model can be considered from the start point of the linear region in Figures 6A,B.

The stress strain response of subject S1 (Figure 6A) showed the decreasing value of strain ( $\varepsilon_{\theta }<0$ ) in the linear region corresponding to 0° angle. However, the stress-strain line for ±90° shows a continuous increment in the strain values ( $\varepsilon_{\theta +90}>0$ ). This can be explained by considering the condition of modulus ratio $E_{\theta }^{L}/E_{\theta \pm 90}^{L}>2$ (Figure 6C). The equal increment in equal biaxial stresses ( $\sigma_{\theta }=\sigma_{\theta +90}$ ) in Eqs 4, 5 caused the decrease in strain ( $\varepsilon_{\theta }<0$ ) corresponding to the direction of higher modulus $\left(E_{\theta }^{L}\right)$ and strain increase $\left(\varepsilon_{\theta \pm 90}>0\right)$ in the direction of lower modulus $\left(E_{\theta \pm 90}^{L}\right)$ . Conversely, for the condition of $\;E_{\theta }^{L}/E_{\theta \pm 90}^{L}<2$ (Figure 6E), the increment in the stress value causes in the increment of strains $\varepsilon_{\theta }>0$ and $\varepsilon_{\theta +90}>0$ for both the directions. The stress strain relation for this condition is illustrated in Figure 6B for subject S2 of group G2, where the angle of 0° and 90° shows continuously increasing strains in the linear region. Similarly, the toe region of both groups falls in the range of the modulus ratio $\;E_{\theta }^{L}/E_{\theta \pm 90}^{L}<2$ . Therefore, the toe region shows similar deformation mechanics to the linear region of group G2 (Figures 6B,E). The modulus ratio $\;E_{\theta }^{L}/E_{\theta \pm 90}^{L}=2$ resulted in zero strain ( $\varepsilon_{\theta }=0)\;$ for the direction of higher modulus as illustrated in Figure 6D. This condition was not witnessed for the maximum and minimum modulus ratio of any subject. However, this condition may occur for the other angles apart from the direction of the overall maximum and minimum modulus ratio.

Figure 6F shows the apparent toe modulus and linear modulus in all the planar directions for the subjects S1 and S2. Our previous study using axially symmetric loading in the bulge test reported a significant correlation between the directional distribution intensity of the collagen fibers and variation in the apparent linear modulus for the skin (Lakhani et al., 2020). This observation indicates that the linear modulus directly represents the directional distribution of the collagen fibers. Therefore, the direction corresponding to maximum and minimum apparent linear modulus (Figure 6F) represents the collagen fibers’ highest and least preferential alignment, respectively. Based on this observation for the *ex vivo* skin, the possible distribution of the collagen fibers for the *in vivo* human skin can be predicted as shown in the schematic diagram of Figures 6G,H for subjects S1 and S2, respectively. These estimated collagens directional distribution intensity can be helpful for the calculation of collagen dispersion and concentration parameters for numerical modeling.

### Effect of Hand Steadiness

**FIGURE 7:**
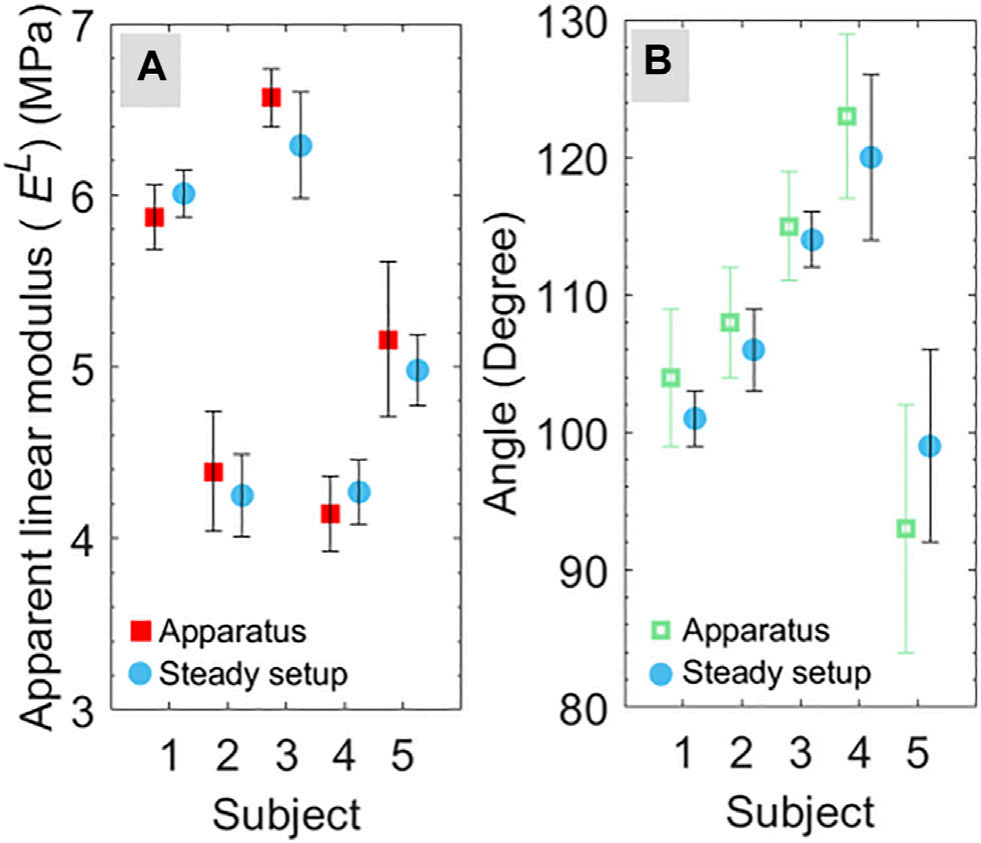
Comparison of **(A)** maximum apparent linear modulus and **(B)** direction corresponding to maximum linear modulus for the test conducted using the apparatus and steady setup for five subjects (from S1 to S5).

The direction of STLs and apparent linear moduli were calculated for five subjects (from S1 to S5) using the steady setup as described in *Materials and Methods*. Then, the apparent linear modulus and orientation of STLs were compared with the results obtained from the developed apparatus. The comparison between the test results of the apparatus and the steady setup is shown in Figure 7. The obtained results do not show a significant difference in the value of apparent linear modulus (*p >* 0.41), and orientation of STLs (*p >* 0.33) obtained using the developed apparatus and steady setup. This observation shows that the stability of the hand for the developed apparatus does not affect the calculated mechanical properties and STLs orientation. Therefore, it can be stated that without compromising measurement accuracy, the developed apparatus is more versatile over the steady experimental setup due to its compactness, portability, and applicability of tests on different body sites. Further, with more compact cameras and inbuilt systems, the developed apparatus can be improved in size and shape.

## Conclusion

The present study developed, validated, and demonstrated a novel full-field measurement suction apparatus that can measure the mechanical stress-strain response for *in vivo* human skin in all the planar directions by a single test. The developed apparatus applies the axially symmetric loading to measure the apparent toe moduli and apparent linear moduli in all planar directions. In contrast, conventionally used systems for measuring elastic modulus require multiple tests to capture the anisotropy (Deroy et al., 2016; Gahagnon et al., 2012; Jacquet et al., 2017; Zhang et al., 2018). Further, the developed apparatus can measure the direction of STLs corresponding to the orientation of the maximum linear modulus. The maximum linear modulus orientation obtained from the apparatus was found in good consistency with the orientation of Kraissl’s lines.

The results demonstrated a significant difference in the toe moduli, linear moduli, and orientation of STLs among the subject. Therefore, it is not realistic to generalize the observation of the present study for a large population. Hence, the developed apparatus can be helpful in the medical application for the subject-specific decision-making processes in the treatment, surgery, and tissue engineering (Terzini et al., 2016) by accurate measurement of STLs orientation and mechanical properties. Further, the measurement of direction dependent variation in the linear modulus can help to estimate the directional distribution intensity of collagen fibers (Lakhani et al., 2020; Lee et al., 2018). This collagen fiber intensity distribution is required for the calculation of concentration parameters and dispersion parameters in the anisotropic numerical modeling of the *in vivo* skin (Gasser et al., 2006). Therefore, this approach may overcome the requirement imaging techniques used to assess collagen fiber dispersion. The imaging technique requires the thin sectioning of the test specimen, which may restrict the collagen dispersion-based numerical modeling of *in vivo* skin.

The working of apparatus mimics the tissue expansion procedure, which is widely applicable in reconstructive surgeries such as traumatic defect repair, burn, hair transplant, and pigmented strains (Pamplona et al., 2014b). In this procedure, nonuniform expansion of skin takes place based on the selection of expander shape and size. Therefore, the understanding of deformation mechanics and anisotropy in the suction test with the different shapes of holes (elliptical, square, and rectangular) can be helpful for the design and optimization of the expander size and shapes (Lee et al., 2018). The optimized shape and size of the expander may reduce the complications, e.g., flap failure, mechanical failure, and implant extrusion (Hodges, 1993). Therefore, future work should be to extend the usefulness of the apparatus for the selection of size and shape of the tissue expander. Also, the study of viscoelastic properties would be possible using the developed apparatus by controlling the strain rate and applying the cyclic deformation on the *in vivo* human skin. Moreover, as a further development of the apparatus, the ultrasound probe (Van Mulder et al., 2017) can be mounted on the apparatus to overcome the limitation associated with the constant thickness assumption.

## Data Availability

The raw data supporting the conclusion of this article will be made available by the authors, without undue reservation.
