# Supplementary material for: Non-Invasive in Vivo Quantification of Directional Dependent Variation in Mechanical Properties for Human Skin
Source: Front Bioeng Biotechnol. 2021 Oct 22;9:749492. doi: 10.3389/fbioe.2021.749492 (PMC8569611; doi:10.3389/fbioe.2021.749492)
Supplement: Supplementary file 1 [file DataSheet1.PDF]

Noninvasive *in vivo* quantification of directional dependent variation in mechanical properties for human skin.

Supplementary Data

1. Steady setup

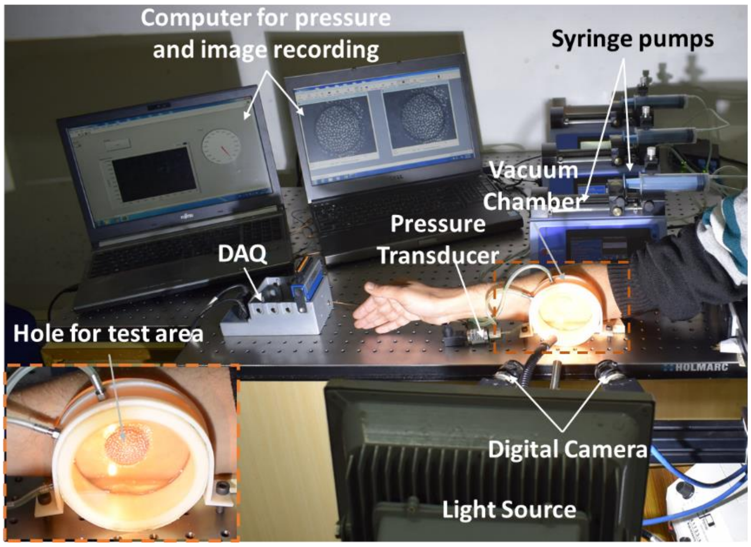

Figure S1. The developed setup used for the assessment of the effect of hand steadiness on measured properties and orientation of STLs.

Figure S1 shows the steady experimental setup developed to understand the effect of hand steadiness for apparatus on measured properties and STLs. A circular frame of the suction chamber having 100 mm outer diameter, 84 mm inner diameter, and 30 mm depth was made of plastic. The front and back covers made of acrylic were rigidly glued with the circular frame to avoid any leakage. Moreover, a circular hole of 30 mm diameter was made on the back cover to put on the skin surface for testing. Suction was created using the syringe pumps (Harvard apparatus, pump 11 elite) and pressure recorded by a pressure transducer through a data acquisition system. The one syringe pump was programmed to withdraw the specified amount of air at a controlled flow rate, and the second syringe pump was attached for safety (to release the vacuum in case of failure in the first syringe pump).

2. Results of uniaxial and suction test performed on silicone substrates.

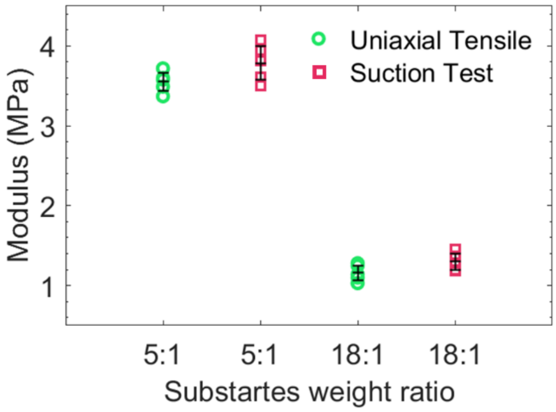

Figure S2. Shows the modulus calculated for the silicone substrates fabricated by mixing the base with a curing agent in a weight ratio of 5:1 and 18:1. Moduli were calculated by taking the linear fit on the stress-strain response obtained from the uniaxial tensile test and suction test.
